# Fish Consumption and Age-Related Macular Degeneration Incidence: A Meta-Analysis and Systematic Review of Prospective Cohort Studies

**DOI:** 10.3390/nu8110743

**Published:** 2016-11-22

**Authors:** Wei Zhu, Yan Wu, Yi-Fang Meng, Qian Xing, Jian-Jun Tao, Jiong Lu

**Affiliations:** 1 Department of Ophthalmology, Changshu No. 2 People’s Hospital, Changshu 215500, China; shzhuwei0722@163.com (W.Z.); meng_yi_fang@163.com (Y.-F.M.); drzheng_gu@163.com (Q.X.); prxuming@163.com (J.-J.T.); 2 Department of Ophthalmology, First Hospital Affiliated to Soochow University, Suzhou 215000, China; txwuyan@suda.edu.cn

**Keywords:** age-related macular degeneration, fish, nutrients, meta-analysis

## Abstract

The association between fish consumption and risk of age-related macular degeneration (AMD) is still unclear. The aim of the current meta-analysis and systematic review was to quantitatively evaluate findings from observational studies on fish consumption and the risk of AMD. Relevant studies were identified by searching electronic databases (Medline and EMBASE) and reviewing the reference lists of relevant articles up to August, 2016. Prospective cohort studies that reported relative risks (RRs) and 95% confidence intervals (CIs) for the link between fish consumption and risk of AMD were included. A total of 4202 cases with 128,988 individuals from eight cohort studies were identified in the current meta-analysis. The meta-analyzed RR was 0.76 (95% CI, 0.65–0.90) when any AMD was considered. Subgroup analyses by AMD stages showed that fish consumption would reduce the risk of both early (RR, 0.83; 95% CI, 0.72–0.96) and late (RR; 0.76; 95% CI, 0.60–0.97) AMD. When stratified by the follow-up duration, fish consumption was a protective factor of AMD in both over 10 years (*n* = 5; RR, 0.81; 95% CI, 0.67–0.97) and less than 10 years (*n* = 3; RR, 0.70; 95% CI, 0.51 to 0.97) follow-up duration. Stratified analyses by fish type demonstrated that dark meat fish (RR, 0.68, 95% CI, 0.46–0.99), especially tuna fish (RR, 0.58; 95% CI, 95% CI, 0.47–0.71) intake was associated with reduced AMD risk. Evidence of a linear association between dose of fish consumption and risk of AMD was demonstrated. The results of this meta-analysis demonstrated that fish consumption can reduce AMD risk. Advanced, well-designed, randomized clinical trials are required in order to validate the conclusions in this study.

## 1. Introduction

Age-related macular degeneration (AMD) is now the leading cause of blindness in developed countries. AMD-related choroidal neovascularization (CNV) or geographic atrophy (GA), in the United States, is expected to increase by 50% by 2020 [1]. Effective treatments, for both early and late AMD, are presently lacking. Major efforts have been made in order to detect the pathogenetic mechanisms of AMD, but the exact etiology of AMD is still unclear [2]. Previous epidemiological studies showed that tobacco smoking was the only consistent causative factor and that other risk factors, such as alcohol consumption and cardiovascular diseases, are inconsistent for AMD incidence or progression [3]. The detections of the potential modifiable factors for AMD incidence would provide better strategies for primary prevention in the future.

As oxidative stress is one of the key pathogenetic factors in the development of AMD, use of antioxidant supplements has been regarded as an effective management strategy of AMD. Antioxidant supplement consumption, including polyunsaturated fatty acids (PUFAs) intake, has been postulated to be a protective factor of AMD [4]. Evidence from cross-sectional [5] and cohort studies [6] demonstrated a significant association between *n*-3 fatty acid consumption and reduced risk of late AMD. In a study of an elderly French population, high concentrations of plasma *n*-3 fatty acids were associated with a decreased risk of late AMD [7]. As we know, the main dietary source of PUFAs is oily fish (e.g., mackerel, tuna, salmon, sardines, and herring) [8], and fish consumption has been reported to be associated with a reduced risk of different types of cancers, diabetes, and several other diseases [9,10]. Based on cross-sectional [11,12], case-controlled [13], and cohort studies [14], fish intake was reported to be associated with a lower risk of AMD. However, there were also a few studies that demonstrated no effect of fish intake on AMD risk. The Eye Disease Case Control Study (EDCC) found no effect of fish intake on incidence for neovascular AMD [15]. In addition, a retrospective analysis of 1968 participants found that fish intake was not associated with AMD incidence compared to less frequent fish consumption [16].

Meta-analyses, which are a useful statistical tool, could pool the relevant, but independent, studies together and, thus, come to a more powerful conclusion. Meta-analysis was also used in the detection of potential risk factors for AMD. For instance, based on a combination of five prospective cohort studies, Chong et al. found that heavy alcohol consumption was associated with an increased risk of early AMD [17]. For these reasons, a meta-analysis and systematic review of the association between fish intake and risk of AMD may help to clarify this issue. The aim of the current meta-analysis was to quantitatively evaluate findings from observational studies on the association between fish consumption and AMD incidence.

## 2. Methods

### 2.1. Search Strategy and Inclusion Criteria

This current study was based on eligible observational studies, and the meta-analysis was conducted according to the Preferred Reporting Items for Systematic Reviews and Meta-Analyses (PRISMA) and Meta-analysis Of Observational Studies in Epidemiology (MOOSE) guidelines [18,19]. A comprehensive search of Pubmed, Embase, and Web of Science was conducted for relevant literature, published up to 15 August 2016, with the combination of “fish”, “seafood”, “life style”, “dietary factor” with “age-related macular degeneration”, “macular degeneration”, “age-related maculopathy”, “maculopathy”, “retinal degeneration”, “drusen”, “choroidal neovascularisation”, and “geographic atrophy”. To acquire all the potential publications, no restrictions were set in the literature search. In addition, the reference lists of relevant articles were also reviewed in order to detect potential eligible studies. If duplicate reports from the same dataset were obtained, only the publications that provided the most comprehensive results were included. If more data from one publication was required, the corresponding author was contacted by e-mail.

The studies that met the following criteria were considered for inclusion in this meta-analysis: (1) the effect of fish consumption on the risk of AMD was reported; (2) results from prospective cohort studies; (3) the values of relative risk (RR) or odds ratio (OR) with 95% confidence intervals (CI) were provided.

### 2.2. Data Extraction and Assessment of Study Quality

Data were independently extracted by two authors (Wei Zhu and Yan Wu) and any disagreements were resolved through discussion with a third author (Yi-Fang Meng). The following data were extracted from each included publication: First author, year of publication, name of cohort, country, age and gender of participants, amount of cases and cohort participants, subtypes or processing methods of fish, adjusting status of the confounding factors, and OR/RR values with 95% CI.

Methodological quality assessment of each included study was assessed by two authors (Wei Zhu and Yan Wu). The assessment scores were checked, and any discord was discussed and a unanimous result was obtained. Considering that all the included studies were cohort studies, the Newcastle-Ottawa Scale (NOS), which was designed for the assessments of observational studies, was used in the assessment of the methodological quality of the included studies [20]. The maximum for NOS was 9 stars and ≥6 stars is considered high quality.

### 2.3. Statistical Methods for the Meta-Analysis

Both OR and RR were extracted from the included studies and used in the final quantitative synthesis. Considering the relative low incidence of AMD, OR values could be used to approximate RR. The adjusted OR/RR values were adopted in the meta-analysis if possible. Both χ^2^ and *I*^2^ methods were used in the assessment of heterogeneity in this study. The inter-study heterogeneity was considered statistically significant if *p* < 0.1 or *I*^2^ > 50%. A random-effects model was used in the estimation of the pooled effects when the inter-study heterogeneity was statistically significant. The effects of fish consumption on AMD risk were delineated with RR and a 95% CI. To conduct sensitivity analyses, we dropped included studies, one-by-one, and observed the modification to the conclusion.

A two-stage, random-effect, dose-response meta-analysis was conducted for the detection of a potential linear relationship between fish consumption and risk of AMD incidence. Restricted cubic splines with four knots, at percentiles of 5%, 35%, 65%, and 95% of the distribution, were used to examine the potential linear dose-response relationship. A *p* value for nonlinearity was detected by testing the null hypothesis that the coefficient of the second spline is equal to 0 [21,22].

Publication bias was assessed using two different methods: Visually evaluating a funnel plot and the quantitative Egger test. A *p* value < 0.05 was regarded as statistically significant. All analyses were conducted with STATA statistical software (version 12.0, Stata Corp LP, College Station, TX, USA).

## 3. Results

### 3.1. Identification and Selection of Studies

**Figure 1:**
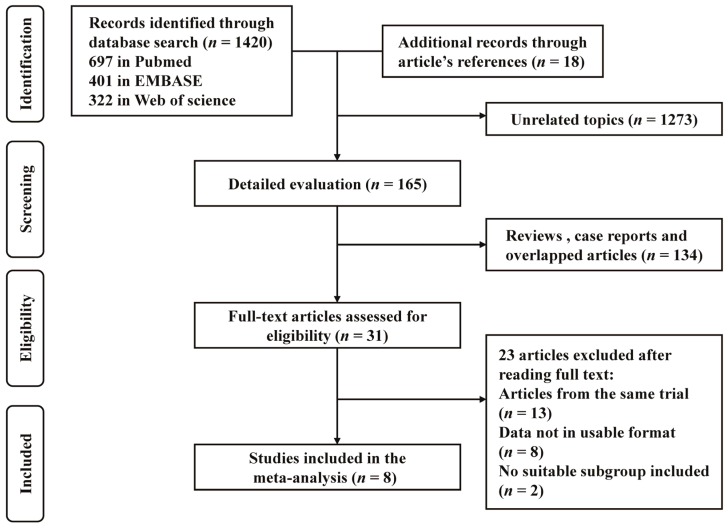
Flow diagram showing the identification of relevant studies in the meta-analysis. The initial 1438 articles were identified, and after 1273 unrelated papers and 134 reviews and case reports were excluded, 31 full texts were assessed for eligibility. Finally, after excluding 23 studies, a total of eight articles were included in this meta-analysis.

A total of 1420 records (697 from Pubmed, 401 from EMBASE, and 322 from Web of Science) were identified through searching the electronic databases. Additionally, 18 more studies were identified through reviewing the reference lists of relevant reviews. A total of 545 unrelated papers were excluded, and 165 publications were reviewed for potential inclusion. After excluding 134 reviews, reviews, case reports, and other articles that reported overlapped data, a total of 31 full texts were assessed for eligibility. Subsequently, a total of 23 studies (13 duplicated studies, eight studies without a usable format, and two studies without conclusive fish intake definitions) were excluded from inclusion, and a final total of eight cohort studies were included for quantitative synthesis [23,24,25,26,27,28,29,30]. The flow diagram is presented in Figure 1.

### 3.2. Study Characters and Quality Scores

A total of 4202 cases with 128,988 individuals from eight cohort studies were identified in this meta-analysis. The detailed characteristics of each included study are presented in Table 1. The included studies were published between 1993 and 2014. In the included studies, drusen, retinal pigment epithelial changes, geographic atrophy, subretinal neovascular membrane, and visual acuity are used in the definition of AMD. Among all the included studies, four studies were in the USA, two in Australia, one in Iceland, and one in the Netherlands. The age, gender distribution, number of cases and cohorts, categories of fish consumption, and adjustments of confounding factors are also demonstrated in Table 1.

The methodological quality of each included study was detected using the NOS scale. NOS was obtained in order to assess the selection, comparability, and outcome of the cohort studies. The scores of each evaluation of all studies are shown in Table 1. All eight included studies were of relatively high quality (over 6 stars) and the mean NOS score was 8.125 stars (standard deviation: 0.295).

### 3.3. Fish Consumption and Risk of AMD

**Figure 2:**
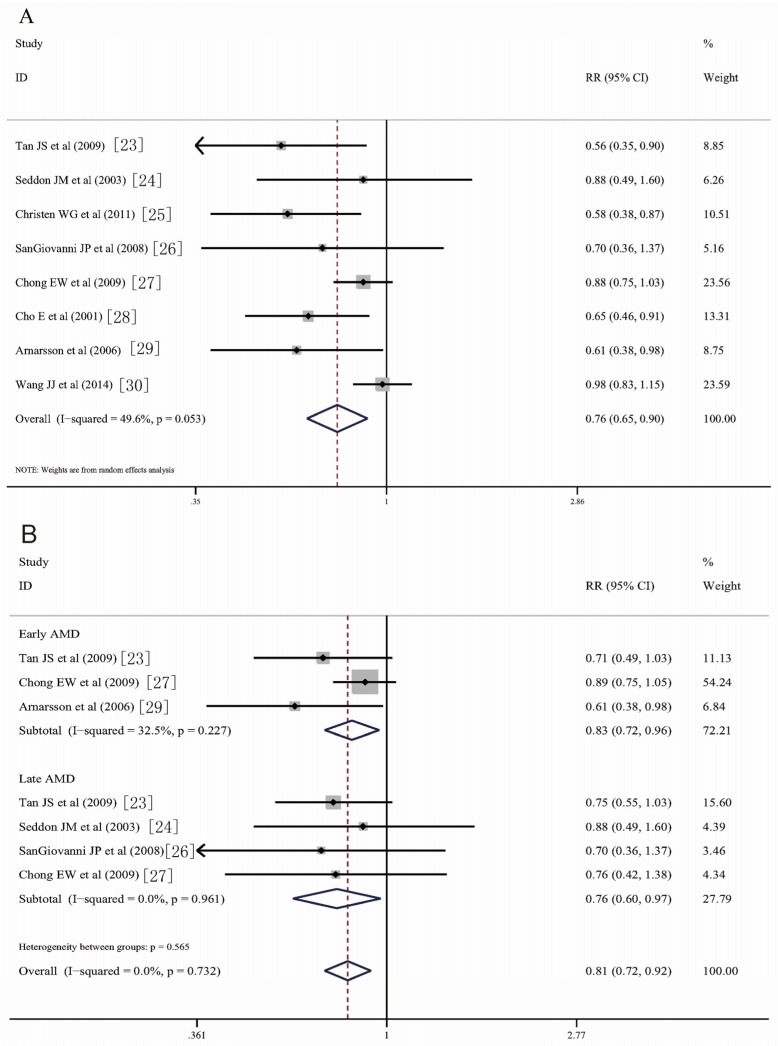
Forest plot of risk estimates of the association between fish intake and risk of age-related macular degeneration (AMD). (**A**) fish consumption and risk of any kind of AMD; (**B**) fish consumption and early and late AMD, through consulting the reference lists of relevant reviews and articles. The size of the shaded square is proportional to the percent weight of each study. Horizontal lines represent 95% confidence intervals (CIs). The diamond data markers indicate pooled odds ratios (ORs).

The pooled estimation on fish consumption and risk of AMD showed that fish consumption can reduce the incidence of AMD. In this meta-analysis, the meta-analyzed RR was 0.76 (95% CI, 0.65–0.90) when any AMD was considered (Figure 2A). Subgroup analysis by AMD stage showed that fish consumption could reduce the risk of both early (RR, 0.83; 95% CI, 0.72–0.96) and late (RR; 0.76; 95% CI, 0.60–0.97) AMD.

Stratified analysis was conducted by data source, study site, and follow-up duration. In the two data source subgroups, a significant association was detected in the population-based group (*n* = 7; RR, 0.75; 95% CI, 0.63–0.89), but not the hospital-based group (*n* = 1, RR, 0.88; 95% CI, 0.49–1.59). When the geographical distributions of the included studies were considered, the studies that were conducted in the USA (*n* = 4; RR, 0.84; 95% CI, 0.72–0.98) and Iceland (*n* = 1; RR, 0.61; 95% CI, 0.38–0.98) showed statistically significant results; however, no significant results were detected in Australia (*n* = 2; RR, 0.74; 95% CI, 0.48–1.14) or in the Netherlands (*n* = 1; RR, 0.98; 95% CI, 0.83–1.15). When stratified by the follow-up duration, fish consumption was a protective factor of AMD in both, over 10 years (*n* = 5; RR, 0.81; 95% CI, 0.67–0.97) and less than 10 years (*n* = 3; RR, 0.70; 95% CI, 0.51–0.97) follow-up durations. All the results of the subgroup analyses are presented in Table 2.

We also detected an association between different types of fish and risk of AMD. It was found dark meat fish (RR, 0.68, 95% CI, 0.46–0.99), especially tuna fish (RR, 0.58; 95% CI, 95% CI, 0.47–0.71) was associated reduced AMD risk. However, no significant association between fish intake and AMD incidence was detected in neither other dark meat fish group (RR, 0.96; 95% CI, 0.75–1.24) nor non-dark meat fish group (RR, 0.82; 95% CI, 0.65–1.03). Subgroup analysis using the processing methods showed that no protective effects were detected in backed, fried or smoked fish group. The results of the detailed stratified analyses are presented in Table 3.

### 3.4. Heterogeneity and Sensitivity Analysis

Heterogeneity was not significant when all eight studies were pooled in the meta-analysis (*I*^2^, 49.6%; *p* = 0.053). When subgroup analysis by AMD subtypes was conducted, no significant heterogeneity was detected in both groups. When the heterogeneity was significant in the subgroup analysis, a random-effects model was obtained to assess the pooled effect.

**Figure 3:**
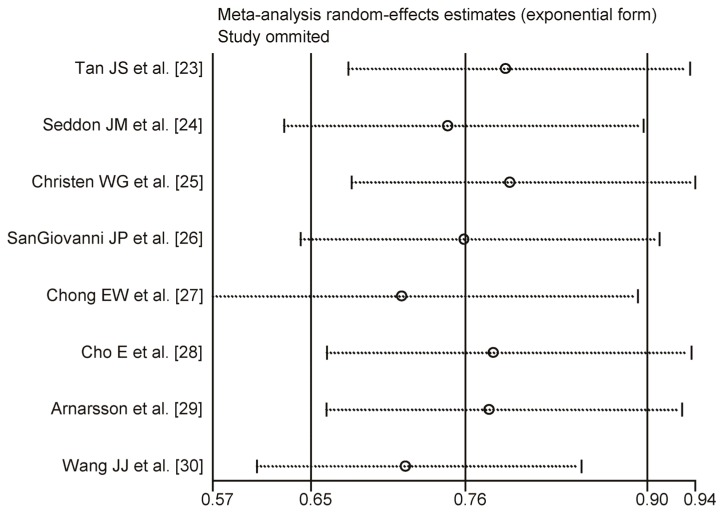
One-way sensitivity analysis for the association between fish intake and AMD risk. There were no studies influencing the result of fish consumption on AMD.

A one-way sensitivity analysis was conducted, and there was little change in the quantitative summary measures of RR or the 95% CI. There were no studies influencing results of fish consumption on AMD. The results of the one-way sensitivity analysis are presented in Figure 3.

### 3.5. Dose-Response Meta-Analysis

**Figure 4:**
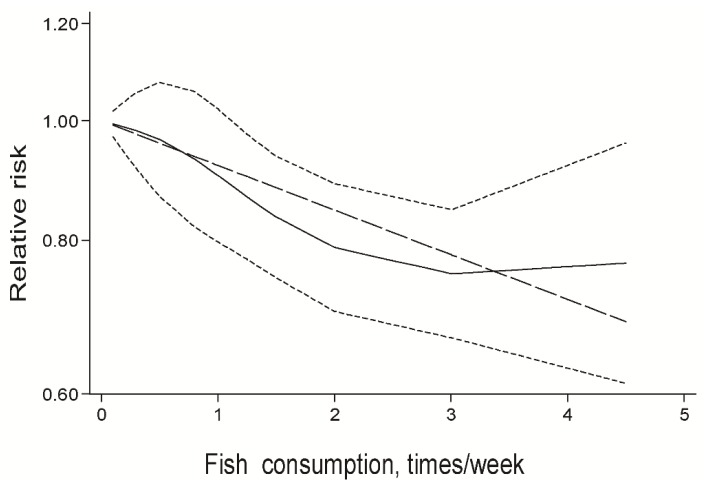
Dose-response relation between fish consumption and relative risks of AMD risk. Lines with short dashes represent the point wise 95% confidence intervals for the fitted nonlinear trend (solid line). Lines with long dashes represent the linear trend.

Considering the significant relationship between fish consumption and the risk of AMD, the potential dose-response relationship was also assessed. It was found that there is a statistically significant association between dose of fish intake and risk of AMD incidence (*p* = 0.001). A one-time-per-week consumption of fish conferred a RR decrease of 0.11 (RR, 0.89, 95% CI, 0.83–0.96; Figure 4).

### 3.6. Publication Bias

**Figure 5:**
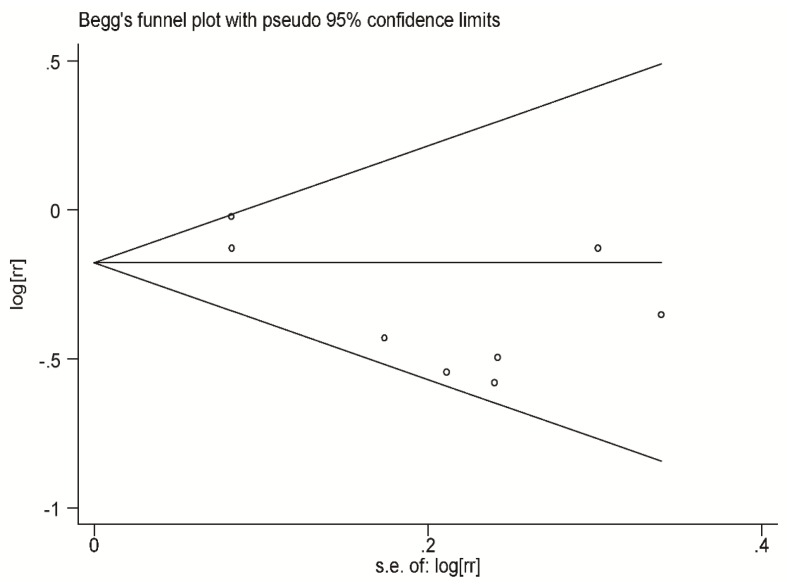
Funnel plot of fish consumption and risk of AMD in the evaluation of publication bias. No significant publication bias was detected through pooling the eight cohorts together.

No significant publication bias in the current meta-analysis was detected using either Begg’s graph or Egger’s test. The funnel plot was symmetrical on visual inspection (Figure 5). In the quantitative assessment, no significant publication bias was detected (Begg’s test, *p* = 0.711; Egger’s test, *p* = 0.068).

## 4. Discussion

A total of 4202 cases with 128,988 individuals from eight cohort studies were identified in this meta-analysis. All the included studies demonstrated a relatively high methodological quality. The findings of this meta-analysis indicated that fish consumption was associated with a reduced risk of AMD. Meanwhile, subgroup analysis by AMD stages showed that fish consumption could reduce the risk of both early and late AMD. When stratified by the follow-up duration, fish consumption was a protective factor of AMD, in both over and less than a 10-year follow-up duration. We also detected an association between different types of fish and risk of AMD. Advanced subgroup analysis showed that dark meat fish and tuna fish showed a protective effect on AMD. In addition, obvious evidence of a statistically significant dose-response relationship between fish intake and AMD risk was detected.

It was reported that inflammation and oxidative stress were key pathologic processes in the development of AMD [31]. Those two examples have long been regarded as potential targets of pharma-projects and primary prevention. PUFAs, which are usually acquired from seafood intake, have been reported to modify the inflammatory reactions and oxidative stress in several diseases [32]. It is natural to presume that additional supplementation of PUFAs would lead to a prevention in the incidence and progression of AMD. Previous epidemiological studies and clinical trials have shown that PUFA supplementation could reduce the risk of AMD [33,34]. However, there were also two studies that reported an increased risk of AMD with higher *n*-3 PUFA consumption [5,35]. A meta-analysis on the association between *n*-3 PUFA intake and AMD risk showed that higher *n*-3 PUFA intake could reduce AMD risk [36]. Plasma *n*-3 PUFA, a nutritional biomarker of *n*-3 PUFA status, was reported to be associated with the incidence of AMD. In a population-based study on nutrition and age-related eye diseases, performed in 963 residents of Bordeaux (France) aged ≥73 years [7], it was found that high concentrations of plasma *n*-3 PUFAs are associated with a decreased risk for late AMD.

Fish, especially tuna fish, is the main source of PUFAs, and higher fish consumption can increase the concentrations of *n*-3 PUFA in blood [37]. In this study, we found that fish consumption could reduce the risk of AMD, and a dose-response effect of fish intake on the incidence of AMD was detected. This result was very consistent with the results from several previous studies. In this meta-analysis, only prospective cohort studies were included. Certainly, case-control studies and cross-sectional studies can provide clues of the related factors of diseases, however, the evidence from cohort studies avoid these types of potential selection biases. The meta-analysis of cohort studies can provide evidence with a higher quality. In the US Twin Study, increased intake of fish reduced risk of AMD, particularly for two or more servings per week [38]. Another multicenter eye disease case-control study involving five US clinical ophthalmology centers showed interesting results; compared with age and sex matched controls, higher fish consumption tended to reduce risk of AMD when the diet was low in linoleic acid. In another case-control study, with 437 advanced AMD cases and 259 unrelated controls, risk of AMD incidence was found to be 51% lower in the highest quartile of fish intake compared to the lowest quartile (OR = 0.49, 95% CI, 0.26–0.90) [39]. Considering that most case-control showed a significant association between fish intake and reduced AMD incidence, it could be presumed that the exclusion of case-control studies in this current meta-analysis would not lead to a significant change in the main outcome.

In this meta-analysis, we found that it was tuna fish but not other types of fish that could reduce the risk of AMD. This was also found in the Nurses’ Health Study (NHS) and the Health Professionals Follow-up Study, a significant inverse association was found only in tuna group. The pooled RR of participants who ate canned tuna more than once 4 times per week was significantly lower (RR, 0.61; 95% CI: 0.45, 0.83) [28]. Tuna fish is rich in PUFAs and it is usually consumed because of its low price. Tuna oil, which is from the muscles of deep sea tuna, contains high concentration of eicosapentaenoic acid (EPA) and docosahexaenoic acid (DHA). A previous cross-sectional study showed that both DHA and EPA consumption was associated with a reduced risk of neovascular AMD [40], while only DHA but not EPA was found to associated with AMD risk in NHS. High DHA content is detected in both brain and retina. Therefore a constant supply of DHA was required for normal regeneration of photoreceptor outer segments and thus produced protective effect in degenerative diseases such as AMD. Increasing evidence showed that the function of DHA on the photoreceptor and retinal degenerative diseases was quite important. DHA is very important on normal conduction in retinal light stimulation. Exogenous DHA helps to keep the fluidity retinal cell membrane. EPA can reduce blood viscosity, dissolve excess fat in the blood vessel wall and reduce blood fat, prevent and improve the effect of cardiovascular. Besides, EPA could help the normal function of DHA in the retina. Moreover, in the consumption of tuna salad, essential fatty acid in tuna salad was mixed vegetable fat and might thus produce more powerful protection in the AMD incidence. Additionally, some other components of tuna fish might affect the incidence or progression of AMD. As we know, different risks modified by fish intake were associated with processing methods. As we see, baked, broiled, fried, and smoked fish intake was not associated with a risk of AMD. We hypothesized that baking, broiling, frying, and smoking processing methods might be harmful to the beneficial materials in fish. In addition, the baked, broiled, fried, and smoked fish processing methods might produce harmful effects for AMD development. Besides, because more significant effect of DHA was detected in mechanism of action and epidemiological features, high DHA/EPA ratio in tuna might explain its particularly stronger inverse association with AMD. Considering few study focused the contribution of DHA/EPA ratio in the AMD progressing, advanced epidemiological studies and experimental studies were required. However, it should be noted that the amount of the publications included in the fish subtype meta-analysis was small and the results in this meta-analysis need to be further confirmed by advanced well-designed study.

Several previous trials were conducted in order to explore the effects of PUFA supplementation on the prevention of AMD. The Nutritional AMD Treatment 2 Study was conducted to evaluate the efficacy of DHA-enriched oral supplementation in preventing exudative AMD [41]. The study was a randomized, placebo-controlled, double-blind, parallel, comparative study, and a total of 263 patients with early AMD lesions and a visual acuity better than 0.4 logarithm of minimum angle of resolution units were included. In wet AMD cases, DHA-enriched supplementation for three years had no significant protective effect on choroidal neovascularization (CNV) incidence in the second eye, as did the placebo. The Age-Related Eye Disease Study 2 (AREDS2) was a multicenter, placebo-controlled RCT in 2006–2012. A total of 4203 participants who were at risk for AMD progression were included in the clinical trial and therapeutic effects of different treatment protocols were compared [42]. It was reported that supplementation of lutein + zeaxanthin, DHA + EPA, or both, failed to further reduce the risk of progression to late AMD. The evidence from the RCTs showed the PUFA supplementation might be not associated with the incidence or progression of AMD. However, the conclusion that fish consumption could reduce the incidence of AMD may not be influenced. Fish is a kind of food with complex components and we cannot exclude the possibility that some other components in fish may also contribute to the association. It should be noted that tuna fish, especially tinned tuna, is an important source of meso-zeaxanthin. Meso-zeaxanthin supplementation has been shown to improve macular pigment optical density in both AMD patients and healthy subjects in a dose-response relationship [43]. In the Meso-zeaxanthin Ocular Supplementation Trial (MOST), it was found that a significant increase in macular pigment from baseline was observed in the meso-zeaxanthin treated group [44]. A previous meta-analysis regarding RCTs showed that *n*-3 PUFA supplementation in people with AMD does not increase the progression or development of AMD [45]. As reported in the SELECT Trial, it was found that men in the highest quartile *n*-3 PUFA level had an increased risk for prostate cancer [46]. It was observed that *n*-3 PUFA supplementation might produce certain harmful effects on chronic inflammation, and a possible explanation for this relates to the fact that polyunsaturated fatty acids act as a substrate for reactive oxygen damage. Dark meat fish, which was the richest source of (docosahexaenoic acid) DHA and (eicosapentaenoic acid) EPA, was associated with reduced AMD risk in this meta-analysis. Thus, additional well-designed studies are required for the detection of the protective effects of anti-oxidants in early AMD.

There are several strengths in this current meta-analysis: (1) A relative comprehensive literature search strategy was used in the search for related publications. We searched databases, including the key words “life style” OR “dietary factor” to detect all available studies; (2) Only prospective cohort studies were included in this meta-analysis, and all included studies demonstrate a relatively high quality. Thus, no significant selection bias influences the conclusion of this study. Robust conclusions were proven through detailed sensitivity analysis and, thus, it suggests that the conclusions of this study are quite credible; (3) A dose-response analysis was conducted and we detected a dose-response relationship between fish intake and AMD risk. The advanced analyses using available data could provide a better understanding of the effect of fish consumption on the risk of AMD.

As with any meta-analysis of observational studies, our study has several limitations. Firstly, the amount of included studies was small. Even through a comprehensive literature search was conducted, only eight studies were included in this meta-analysis. This limited the dependability of subgroup analysis, as only a few studies were included. Secondly, most studies did not provide data stratified by some important confounding factors, such as tobacco smoking and family history. Although all the RR values of the included studies were adjusted by key factors, the influence of these factors should not be ignored. These points all indicate the requirement of additional well-designed studies in the future.

## 5. Conclusions

In conclusion, the results from this meta-analysis of prospective cohort studies demonstrated that fish consumption, especially tuna fish, could reduce AMD incidence. There was a significant dose-response relationship between fish consumption and risk of AMD. However, additional longitudinal studies with more detailed data, such as fish subtypes or processing methods, are still required and would provide a better understanding on this issue.

## Figures and Tables

**Table 1:** Characteristics of eligible studies.

Author, Year	Study; Follow-up	Duration	Study Design	Site	Age (Year)	Gender, Percent	No. of Case/Cohort	Adjustments of Confounding Factors	Question	Exposure Definition	Study Quality *
Tan et al., 2009 [23]	Melbourne Collaborative Cohort Study > 10 years	1992–2004	Population-based	Australia	≥49	F: 57%	232/2684	Age, sex, and smoking	145-item FFQ	<1/M (Q1) vs. ≥3/W (Q3)	8
Seddon et al., 2003 [24]	AREDS, 4.6 years	1989–1998	Hospital-based	USA	≥65	F: 61%	51/312	Age-sex group, education, body mass index, systolic blood pressure, cardiovascular disease, log energy, protein intake, energy-adjusted log beta carotene intake, alcohol intake, physical activity, and initial age-related macular degeneration grade, total intake of energy-adjusted log zinc, vitamin C, and vitamin E.	61-item FFQ	<1/W (Q1) vs. ≥2/W (Q3)	8
Christen et al., 2011 [25]	Women’s Health Study, 10 years	1993–2004	Population-based	USA	≥45	F:100%	235/38257	Age, randomized treatment assignment, smoking, alcohol use, BMI, menopausal status and use of HT, history of hypertension, history of high cholesterol, history of diabetes multivitamin use, history of eye exam in the last 2 years	131-item FFQ	<1/M (Q1) vs. >1/M (Q3)	7
SanGiovanni et al., 2008 [26]	Massachusetts Eye and Ear Infirmary, 6.3 years	1992–1998	Population-based	USA	55–80	F: 56.1%	311/2623	Age, sex, AREDS therapy group, education, race, BMI, smoking, antacid use, iris colour, DHA intake, EPA intake, combined DHA-EPA intake	90-item FFQ	<1/M (Q1) vs. >2/M (Q5)	9
Chong et al., 2009 [27]	Nurses’ Health Study, 13 years	1990–2006	Population-based	Australia	66–85	F: 61%	1099/7098	Age, sex, smoking (current, past, or never), energy, vitamin C, vitamin E, carotene, zinc, lutein, zeaxanthin, and supplements (vitamin C, vitamin E, cod liver oil and fish oil (yes/no))	121-item FFQ	0–0.5/W (Q1) vs. ≥2/W (Q3)	9
Cho et al., 2001 [28]	Blue Mountains Eye Study, 12 years	1984–1996	Population-based	USA	56	F: 59.0%	567/73056	2-year period, age , smoking, energy and lutein and zeaxanthin intakes, BMI, profession, physical activity (metabolic equivalent quintiles), and alcohol intake	130-item FFQ	≤1/M (Q1) vs. ≥4/W (Q5)	9
Arnarsson et al., 2006 [29]	Reykjavik Eye Study, 5 years	1996–2001	Population-based	Iceland	≥50	F: 55.8%	134/1379	Age, smoking, and sex	16-item FFQ	≤1/M (Q1) vs. ≥4/W(Q4)	7
Wang et al., 2014 [30]	Rotterdam Study, 15 years	1990–2001	Population-based	The Netherlands	≥55	F: 58.8%	1573/3579	Age- and sex-adjusted	170-item FFQ	<1/W (Q1) vs. ≥1/W(Q2)	8

F: Female; BMI: Body mass index; HT: Hormonal therapy; FFQ: Food frequency questionnaire; AREDS: Age-Related Eye Disease Study; DHA: Docosahexaenoic acid; EPA: Eicosapentaenoic acid. *: The study quality was assessed independently by two reviewers using the Newcastle-Ottawa scale (NOS). The maximum of NOS was 9 stars for a study, and a study with over 6 stars was regarded as being of relatively high quality.

**Table 2:** Subgroup analysis of fish consumption and risk of AMD with combined relative risks (RR).

Subgroups	No. of Studies	Summary Effect		Study Heterogeneity	
		RR (95% CI)	*p* Value	*I*^2^, %	*p* Value
Data source					
Population based	7	**0.75; (0.63–0.89)**	**0.001**	56.7	0.031
Hospital based	1	0.88 (0.49–1.59)	0.672	-	-
Country					
USA	4	**0.84 (0.72–0.98)**	**<0.001**	0	0.724
Australia	2	0.74 (0.48–1.14)	0.174	68.50	0.075
Iceland	1	**0.61 (0.38–0.98)**	**0.002**	-	-
Netherlands	1	0.98 (0.83–1.15)	0.787	-	-
Follow-up					
>10 years	5	**0.81 (0.67–0.97)**	**0.024**	53.6	0.072
< 10 years	3	**0.70 (0.51–0.97)**	**0.033**	0	0.638

AMD: age-related macular degeneration. RR: Relative risk; CI: Confidence interval. The result in bold demonstrate a significant outcome.

**Table 3:** Stratified analysis of fish subtypes and processing methods and risk of AMD with combined RR.

Subgroups	Summary Effect				Study Heterogeneity	
	RR	95% Lower Limiter	95% Upper Limiter	*p* Value	*I*^2^, %	*p* Value
Fish types						
**Dark meat fish**	**0.68**	**0.46**	**0.99**	**0.047**	**53.70**	**0.091**
**Tuna fish**	**0.58**	**0.47**	**0.71**	**<0.001**	0	0.934
Other dark meat fish	0.96	0.75	1.24	0.34	-	-
Non-dark meat fish	0.82	0.65	1.03	0.088	0.80	0.315
**Processing**						
Baked or broiled	0.98	0.87	1.11	0.762	0	0.488
Fried fish	0.97	0.83	1.14	0.731	0	0.508
Smoked fish	0.88	0.54	1.43	0.600	0	0.974

RR: Relative risk. The results in bold demonstrate a significant outcome.
